# Stress and autonomic response to sleep deprivation in medical residents: A comparative cross-sectional study

**DOI:** 10.1371/journal.pone.0214858

**Published:** 2019-04-04

**Authors:** Jose Morales, Alexandre Yáñez, Liria Fernández-González, Lluïsa Montesinos-Magraner, Adrià Marco-Ahulló, Mónica Solana-Tramunt, Esther Calvete

**Affiliations:** 1 Faculty of Psychology, Education Sciences and Sport Blanquerna, Ramon Llull University, Barcelona, Spain; 2 Department of Personality and Psychological Assessment and Treatment, University of Deusto, Bilbao, Spain; 3 Unidad de lesionados medulares, Vall d'Hebron Research Institute, Barcelona, Spain; University of Rome Tor Vergata, ITALY

## Abstract

The aim of this study was to evaluate the stress suffered by medical residents as the result of being on call for 24 hours, from a multidimensional approach. Two groups of medical residents selected according to their work shift, participated in the study: one group (*n* = 40) was sleep-deprived after having been actively on-call for 24 hours, and another contrast group (*n* = 18) had performed a normal work day and were not sleep-deprived. All participants completed pre-post measures during a 24 h cycle. These were administered on both occasions at 8 am. The measures included HRV, cortisol, cognitive performance and transitory mood. The effect of the group x phase interaction was significant for all variables analysed, indicating that doctors in the 24h on-call shift group showed significant deterioration in all physiological, performance and mood indicators in comparison with the participants in the group not on call. These results suggest the need to review medical on-call systems, in order to reduce the stress load, which has a direct effect on working conditions.

## Introduction

Lack of sleep is a widespread problem in today’s society, and it can negatively affect psychological and physiological functioning [1]. At the psychological level, lack of sleep is accompanied by adverse changes, such as symptoms of anxiety and depression [2], reduced cognitive performance and the deterioration of judgement capacity [3]. At the physiological level, deterioration has been found in the immune function [4], hormonal alterations [5] and increased cardiovascular risk [6].

The most common causes of sleep deprivation include work-related factors. In this context, health professionals are especially prominent, who in addition to inadequate sleep, also suffer from stressful circumstances, such as time pressure, high expectations, low tolerance for errors and, in particular, night-time on-call work [7]. There is some controversy about the appropriateness of long work days that demand the participation of medical residents who are on call for 24 hours. On the one hand, the results of an important number of studies question this system, arguing that it leads to more medical errors that jeopardise the safety of patients [8–10]. On the other hand, there are also studies showing that participation in 24h on-call shifts has benefits for the training of medical residents and does not jeopardise patient safety [11,12].

The impact of 24h on-call shifts on healthcare professionals has been studied from different perspectives, including performance, emotional and physiological effects. In general, it has been found that sleep deprivation causes a deterioration in attention, state of alertness and executive functions in general, as well as an increase in blood pressure, stress hormone levels and a reduction in parasympathetic tone [13]. From the perspective of the performance of healthcare workers after 24h on-call shifts, the main studies have focused on simulating clinical tasks [14], attention and monitoring tasks [15], and performance of cognitive tasks [8,16]. Other studies have examined the effect of sleep deprivation on doctors by means of questionnaires such as the d2 Test [17] and the Continuous Performance Test [13], showing altered attention and working memory, both of which are susceptible to causing errors in professional tasks.

From the perspective of the impact observed on the mental health and emotions of medical residents after 24h on-call shifts, several studies have found negative effects of sleep deprivation on variables such as burnout, perceived stress [18,19] and anxiety [20,21].

Finally, from a physiological perspective, studies have examined indicators of activation of the autonomous nervous system (ANS). Analysis of heart rate variability (HRV) is a very useful tool for monitoring ANS. HRV reflects time oscillations in the interval between heartbeats (R-R) and is a non-invasive method used to assess the interactions between the sympathetic and parasympathetic systems [22]. Moreover, the reduction in HRV and vagal tone is associated with higher levels of anxiety [23,24], and some studies have indicated that the reduction in frequency-domain HRV could be related to a lack of sleep and insomnia [19,25]. For example, Amirian et al. [26] found that surgeons who worked night shifts presented a significant decrease in HRV and a significant increase in heart rate, which represented an increase in the sympathetic domain in the ANS. In another study, HRV was compared in nurses grouped according to the stress shown at work and the results demonstrated that the persistence of stress reduces the parameters in the temporal domain of HRV, which supports the hypothesis that ANS disorders can contribute to the relationship between work-related stress and cardiovascular disorders [27].

The previous review shows that the adequacy of medical residents participating in active 24h on-call shifts is still a topic that is open for debate [10,12]. One of the limitations of the state of the question is that studies evaluating the impact of 24h on-call shifts from a multidimensional perspective, including HRV parameters, subjective measures and cognitive performance, are still relatively scarce and their results are mixed. It is therefore necessary to delve deeper into this area in order to clarify the impact of night-time on-call shifts on medical residents in terms of the multiple components of stress.

The present study was conducted to evaluate the impact of 24h on-call shifts on medical residents from a multidimensional perspective, using indicators in different areas: physiological condition (HRV and cortisol), mood and cognitive performance. The main objective was to compare a group of medical residents who completed an intensive 24h on-call shift with sleep deprivation and another group that completed a normal work day without sleep deprivation in terms of HRV, cortisol, cognitive performance and transitory mood. We hypothesised that the sleep deprivation group would present evidence of significantly greater deterioration on a physiological, subjective and cognitive level. A secondary objective consisted of examining the association between these different dimensions of stress response.

## Materials and methods

### Participants

In 2017, a total of 58 medical residents who work at two public hospitals belonging to the Catalan Health Institute, Hospital Universitari Vall d’Hebron (Barcelona) and Hospital Universitari Germans Trias (Badalona) participated in this study. The distribution by gender was 31 women and 29 men, with a mean age of 30.33 ± 4.18 years. The average height was 167 ± 9 cm, body mass was 69.47 ± 13.20 kg, mean body mass index (BMI) was 25.35 ± 2.87 kg·m^2^, and their mean work experience as medical residents was 4.3 ± 2.6 years. The objectives and procedures of the study were explained and all subjects signed the informed consent form. This study was approved by the Ethics and Research Committee at Blanquerna Faculty of Psychology and Educational and Sports Sciences of Ramon Llull University with the reference number 1516002P. The inclusion criteria were as follows: having more than one year of experience as a medical resident, not taking any medication at the time of data collection, not having any disease or sleep disorder and, for female participants, not being pregnant or in the follicular phase of menstruation. Furthermore, in the case of the control group, an additional inclusion criterion required that the night before the post-test the participant had slept a maximum of one hour more or less than his or her normal average.

All protocols applied in this research (including the management of the personal data of the participants) complied with the requirements specified in the Declaration of Helsinki of 1975 and its subsequent revisions.

### Design

This is a comparative cross-sectional study of two groups with two repeated measures (pre- and post-test) during a 24h cycle, administered on both occasions at 8 am. The groups were selected according to their work shift: participants in the first group (*n* = 40) were sleep-deprived after having been actively on-call for 24 hours, whereas participants in the contrast group (*n* = 18) had a normal work day and were not sleep-deprived. This design made it possible to compare the results between a normal work day and an active 24h on-call work day. The dependent effects of circadian rhythms were minimised by evaluating the participants at the same time of day. All participants were asked to abstain from consuming alcohol and caffeine during the study and for 24h hours prior to data collection. Data was collected during a period of approximately 20–25 minutes, focusing on the following tests: physiological measures (HRV and salivary cortisol), psychological measures (Mood Assessment Scale—EVEA) and cognitive performance (Psychomotor Vigilance Test—PVT). 24 hours passed between the two measurements. The first measurement was taken at 8.00 a.m. (at the beginning of the working day) and the second at 8.00 a.m. the following day (beginning of the working day for the control group and the end of the shift for the experimental group).

### Measures

HRV recording was programmed considering that the female participants were not in the follicular phase of menstruation, as it has been shown that there is a decrease in parasympathetic activity and an increase in sympathetic activity in this phase as compared to the luteal phase [25]. HRV data was recorded for a period of 10 minutes. The temperature of the location where the measurements were taken varied from 20–22° C and no noise or anything else was perceived that could alter proper data collection. All participants remained in a prone supine position in absolute silence, in a relaxed state and with slow, paused breathing (10–12 cycles/min) at the start of the pulse recording.

For HRV data collection, an RS800 Polar pulsometer and a Polar Electro Oy (Kempele, Finland) encoded transmitter were used to record the RR (beat to beat) signal. This material has been validated for HRV measurements [28]. The analysis was carried out using the last 5 minutes of recording, as the first 5 minutes were used to establish the heart rate and were ultimately discarded from the analysis. RR interval data files were transferred to a computer using Polar-specific software (PolarProtrainer 5, Polar Electro, Kempele, Finland). Further signal processing was performed using a dedicated HRV analysis program (Kubios HRV Analysis version 2.0, The Biomedical Signals Analysis Group, University of Kuopio, Finland). Occasional ectopic beats were automatically replaced with the interpolated adjacent R–R interval values.

The data was analysed according to the criteria established by the Task Force of the European Society of Cardiology and the North American Society of Pacing and Electrophysiology [22]. The values used from the temporal domain were those corresponding to the square root of the mean value of the sum of the squares of differences between R-R (RMSSD), which is a clear example of parasympathetic modulation [29]. As recommended by the Task Force [22] the spectral response was decomposed into three bands: Very Low-Frequency (VLF), Low-Frequency (LF) and High-Frequency (HF). The interpretation of VLF is highly doubtful, and its use is therefore not recommended [22,30]. However, there is consensus regarding the interpretation that HF is under parasympathetic control, LF reflects both components of sympathetic and parasympathetic control and the LF/HF ratio is a measure that estimates sympathetic-vagal balance [31]. In this study, the temporal domain values were calculated based on the Fast Fourier Transform (FFT) function, which quantified the spectral density of the LF (0.04 to 0.15 Hz) and HF (0.16 to 0.40 Hz) bands. The calculation of the LF/HF ratio was also included.

The saliva samples for cortisol analysis were taken with Salivette tubes (Sarstedt, Nümbrecht, Germany). These devices were centrifuged and stored at -25° C immediately after collection. Cortisol was measured by an automated electrochemiluminescence assay (ECLIA) (Roche Diagnostics GmbH, Mannheim, Germany). The sensitivity of the assay was 0.054 ug/dL, and the interassay coefficient of variation at a mean concentration of 0.34 ug/dL was 7.1%.

To evaluate the participants’ mood, the Mood Assessment Scale (EVEA) [32] was used, which is an instrument designed to evaluate four situational emotional states of a clinical nature: depression, anxiety, hostility and happiness. This scale consists of 16 items based on adjectives that define moods, which are scored on a Likert scale (0–10). The items are grouped into four moods that are represented by the four subscales of the EVEA. This instrument is used to evaluate the changes in mood that occur over short periods, for example before and after a work day. The Cronbach’s alpha coefficients ranged from .88 to .93.

Cognitive performance was evaluated by means of PVT, which is a test based on reaction time (RT) to stimuli that are produced randomly, and thus it also measures level of attention. It has frequently been used to objectively evaluate the degree of cognitive deterioration related to sleep loss [33]. It has shown good reliability, with an ICC of 0.8, as measured by a test-retest. The standard version of a 10-minute PVT was proposed by [34]. The resulting measurements were the response speed represented by the mean RT and number of lapses (responses > 500ms) and the number of anticipated responses (responses < 100ms). A 15” laptop computer with specific E-Prime v2 (Psychology Software Tools, Inc. Pittsburg PA) software was used to administer the test, presenting the stimuli and processing the responses. Participants were instructed to press the response button when a square with horizontal stripes appeared onscreen. The random duration of the intervals between stimuli was 2–10 seconds. The subjects were also told that they were not allowed to respond ahead of time or delay their response, or respond if the stimulus was a square with vertical stripes; in these cases, the response would be counted as an error.

### Statistical analysis

All descriptive data from the dependent variables is presented with the mean ± standard deviation (SD). The normal distribution of each variable was checked with a Kolmogorov-Smirnov test. All variables met normalcy criteria, except for the variable RMSSD. In this case, the natural logarithm (Ln) was calculated for each so that they met normalcy criteria.

Pearson’s correlations were used to study the association between the different parameters (HRV, Cortisol, EVEA and PVT) in pre-test and post-test situations. The relationships between variables were evaluated with the following thresholds [35]: <0.1 trivial, 0.1 to 0.3 small, > 0.3 to 0.5 moderate, > 0.5 to 0.7 large, > 0.7 to 0.9 very large and > 0.9 to 1.0 almost perfect.

In order to test the study hypotheses, a mixed model analysis of variance (ANOVA) was applied [Phase (2: PRE, POST) × Group (2: Control, Experimental) × Gender (2: Men, Women)], including the physiological, subjective and cognitive performance measurements as dependent variables. In the analysis of the physiological variables, the BMI was controlled as a covariate to determine whether there was any type of influence. Bonferroni’s test was applied for pairwise comparisons. The level of significance was set at *p* < 0.05 in all analyses. All the statistical analyses were calculated using the Statistical Package for Social Science version 22.0 software (SPSS, Inc., Chicago, IL, USA).

## Results

### Descriptive statistics

Tables 1 and 2 show the correlation coefficients between them at pre-test and post-test, respectively. In the pre-test, all the physiological parameters significantly correlated with one another, with effect sizes that ranged from moderate to high; except for the LF/HF ratio, which was not significantly associated with any variable. The subjective measures were significantly associated in the expected direction with the cardiovascular parameters. The cognitive performance indicators were not significantly associated with the subjective and cardiovascular variables. The exceptions were the correlation coefficients between anticipated responses and cortisol and anxiety, which were statistically significant.

**Table 1:** Correlation coefficients between the study variables in the pre-test.

	1	2	3	4	5	6	7	8	9	10	11	12
1. LnRMSSD												
2. LF/HF ratio	-.23											
3. LF	-.83***	.01										
4. HF	-.91***	.17	.89***									
5. Cortisol	-.84***	-.08	.80***	.77***								
6. Depression	-.49***	.07	.57***	.44**	.52***							
7. Anxiety	-.72***	.03	.74***	.75***	.77***	.50***						
8. Hostility	-.26*	.03	.47**	.46***	-.26*	.18	.57***					
9. Happiness	.45***	.09	-.49***	-.41**	-.55***	-.78***	-.49***	-.28*				
10. RT	-.18	.09	-.06	.09	.02	-.16	-.21	-.24	.12			
11. Errors	.02	-.19	-.01	-.06	.08	-.02	.04	-.07	.01	.19		
12. Lapses	.10	-.13	-.09	-.18	-.06	.02	-.10	-.11	-.00	.12	.90***	
13. Anticipated responses	-.19	-.16	.16	.15	-.31*	-.10	-.31*	.08	.04	.16	.33**	-.10

Note: RMSSD = Square root of the mean squared difference of successive RR interval; LF = Low-Frequency; HF = High-Frequency; RT = Reaction time.

* *p*< .05

** *p* < .01

*** *p* < .001.

**Table 2:** Correlation coefficients between the study variables in the post-test.

	1	2	3	4	5	6	7	8	9	10	11	12
1. LnRMSSD												
2. LF/HF ratio	-.28*											
3. LF	-.73***	.15										
4. HF	-.91***	.35*	.89***									
5. Cortisol	-.63***	.11	.87***	.72***								
6. Depression	-.10	-.03	.37**	.21^a^	.37**							
7. Anxiety	-.63***	.14	.84***	.65***	.84***	.47***						
8. Hostility	-.35**	.48**	.46***	.43***	.40**	-.31*	.55***					
9. Happiness	.47***	-.05	-.65***	-.53***	-.61***	-.46***	-.83***	-.41**				
10. RT	.27*	.14	-.29*	-.26*	-.35**	-.17	-.29*	.22	.35**			
11. Errors	.42**	.25	-.37**	-.42**	-.35**	-.09	-.18	.31*	.13	.69***		
12. Lapses	.40**	.22	-.35**	-.48**	-.32*	-.08	-.17	.33*	.12	.68***	.98***	
13. Anticipated responses	.19	.03	-.18	-.15	-.20	-.04	-.08	-.02	.04	.23^a^	.32*	.12

Note: RMSSD = Square root of the mean of the sum of the squared differences of all the successive RR intervals; LF = Low-Frequency; HF = High-Frequency; RT = Reaction time.

* *p* < .05

** *p* < .01

*** *p* < .001.

The pattern of correlations between variables in the post-test was fairly similar to that obtained in the pre-test, with some notable exceptions. The LF/HF ratio was significantly correlated to the S/PS ratio and hostility. Three cognitive performance indicators (RT, errors and lapses) were significantly correlated to RMSSD, SS, S/PS ratio and cortisol. In addition, the hostility score was significantly associated with the number of errors and lapses. Finally, the experimental and control groups were compared for the sociodemographic variables of gender and age, with no statistical differences found: gender, χ^2^_(1)_ = 0.16, *p* = .69; and age, *t*_(51)_ = 0.89, *p* = .38.

### Comparison between groups

**Fig 1:**
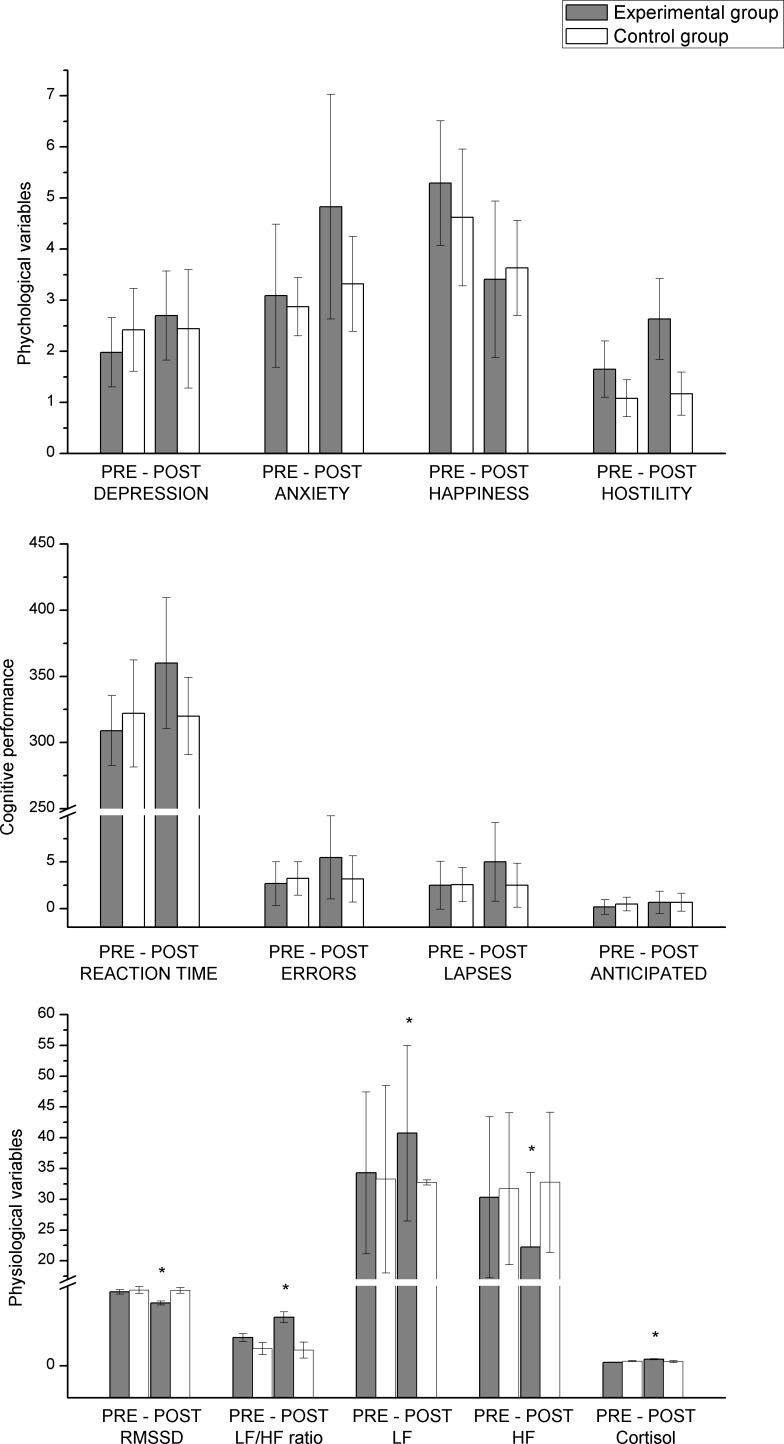
Pre-post test scores for the variables by group.

Table 3 shows the means and standard deviations for the dependent variables studied (physiological, psychological and cognitive), for the pre-test and post-test phases, in each of the study groups (i.e., experimental group– 24h active on-call work day–and control group–normal work day). This table also displays the ANOVA results for the interaction between phase and group. As can be seen, the effect of the group x phase interaction was significant for all physiological variables analysed, with effect sizes (η^2^_p_) varying between .07 and .35. Participants in the experimental group showed a significant deterioration in all the physiological indicators analysed, in comparison with the participants from the contrast group. The largest effects were found for RMSSD values and cortisol levels (see Fig 1). The triple interaction of group x phase x gender was not significant, which indicates that the effect of the 24h active on-call work day was similar for both men and women. No influence of the BMI co-variable on the physiological variables was found.

**Table 3:** Pre- and post-test means and standard deviations for experimental and control groups.

	Experimental group			Control group					
		Pre-test	Post-test		Pre-test	Post-test			
Variables	*n*	M (S*D*)	M (S*D*)	*n*	M (S*D*)	M (S*D*)	*F-value*	*p*-value	*η*_*p*_^*2*^
**Physiological**									
LnRMSSD (ms)	40	4.63 (0.14)	3.93 (0.12)	18	4.73 (0.21)	4.72 (0.18)	28.19	< .001	.35
LF/HF ratio (ms^2^)	40	1.76 (0.25)	3.04 (0.34)	18	1.07 (0.38)	0.99 (0.50)	4.16	.046	.07
LF (ms^2^)	40	34.28 (13.14)	40.73 (14.25)	18	33.27 (15.24)	32.71 (11.66)	9.61	.005	.16
HF (ms^2^)	40	30.31 (13.09)	22.25 (12.11)	18	31.71 (12.34)	32.75 (13.55)	15.34	< .001	.23
Cortisol (ug/dL)	40	0.22 (0.02)	0.41 (0.04)	18	0.29 (0.03)	0.26 (0.06)	16.22	< .001	.23
**Psychological**									
Depression	40	1.98 (0.68)	2.70 (0.87)	18	2.42 (0.81)	2.44 (1.16)	8.23	.006	.13
Anxiety	40	3.09 (1.40)	4.83 (2.20)	18	2.87 (0.57)	3.32 (0.93)	8.50	.005	.13
Happiness	40	5.29 (1.22)	3.41 (1.53)	18	4.62 (1.34)	3.63 (0.93)	3.98	.051	.07
Hostility	40	1.65 (0.55)	2.63 (0.79)	18	1.08 (0.36)	1.17 (0.42)	27.39	< .001	.33
**Cognitive performance**									
RT (ms)	40	309 (26.36)	360 (49.46)	18	322 (40.50)	320 (29.14)	19.12	< .001	.26
Errors (number)	40	2.67 (2.34)	5.48 (4.46)	18	3.22 (1.80)	3.17 (2.48)	6.70	.012	.11
Lapses (number)	40	2.50 (2.56)	5.00 (4.21)	18	2.56 (1.85)	2.50 (2.36)	7.13	.010	.12
Anticipated responses	40	0.17 (0.79)	0.67 (1.19)	18	0.48 (0.74)	0.67 (0.97)	1.19	.280	.02

Note: RMSSD = Square root of the mean of the sum of the squared differences of all the successive RR intervals; LF = Low-Frequency; HF = High-Frequency; RT = Reaction time.

With regard to subjective variables, the group x phase interaction was statistically significant for all variables, except for the happiness variable, for which the effect was marginally significant (*p* = .051). As shown in Table 3, the participants from the experimental group experienced a greater increase in scores for negative emotions after the work day than the participants in the contrast group did. The greatest effect size was observed for hostility. Fig 1 shows the changes between pre-test and post-test for both groups for hostility. The triple interaction between group x phase x gender was not statistically significant for any subjective variable.

Finally, with regard to cognitive performance, the interaction between group x phase was statistically significant for RT, number of errors and number of lapses. The greatest effect size was observed for RT. Fig 1 shows the results of the interaction for RT. The group x phase x gender interaction was not statistically significant for any cognitive performance variable.

## Discussion

The main aim of this study was to evaluate the stress suffered by medical residents as the result of being on call for 24 hours, from a multidimensional approach. The use of endocrine, cardiovascular, cognitive and mood parameters revealed the expected results, indicating an increase in stress after a 24h on-call shift that was significantly higher in comparison with another group of medical residents who completed a normal work day without sleep deprivation.

The comparison between groups shows the magnitude of the stress caused by 24h on-call shifts and the important physiological effect on the participants’ bodies, without taking into account gender, since no significant differences were observed in any case between men and women. Our findings show that the participants who worked night-time on-call shifts experienced reduced HRV and increased levels of basal cortisol, which could put them at greater risk of cardiovascular diseases [29]. Previous studies such as Tobaldini et al. [36] found reductions in HRV in response to orthostatic stress, but not in the cortisol levels in physicians after one night on-call. Meanwhile, the meta-analysis performed by Capuccio et al. [37] also described the association between short sleep duration and increased cardiovascular risk. Other short-term sleep deprivation studies have shown that the variables of Systolic Blood Pressure, Diastolic Blood Pressure and Heart Rate all tend to increase the day after a sleep-deprived night, associated with a significant increase in plasma and urinary catecholamine levels and LF/HF ratio, suggesting that sleep deprivation leads to a sympathetic-parasympathetic imbalance, with a prevalence of sympathetic modulation [37,38].

In our case, the significant reduction in HRV in the experimental group in comparison with the control group indicated that the stress was intense enough to inhibit the vagal modulation of ANS in the experimental group. However, the control group experienced no significant changes in HRV between the pre- and post-tests. HRV measurements were quantified in three analysis domains [22], indicating in all cases an increase in sympathetic activity and a modulation of parasympathetic activity, reflected by an overall reduction in HRV. Relatively few studies have examined HRV together with other stress-related parameters (e.g., mood, biomarkers and performance of cognitive tasks) to study the impact of 24h on-call shifts on medical residents. The few studies that have been conducted show mixed results. For example, Shen et al. [20] compared the levels of insomnia, anxiety and HRV in 114 nurses who worked fixed shifts with those who carried out rotating on-call shifts. A greater effect on anxiety levels and greater modulation of the activity of the sympathetic SN, both related to altered sleep patterns, was found in the group that worked rotating shifts. Nevertheless, studies with similar designs did not examine all the HRV parameters analysed in the present research. Specifically, Martinez de Tejada et al. [39] looked at a sample of 18 medical residents at a hospital in Geneva (Switzerland) and found a significant increase in the levels of such biomarkers as adrenalin and dopamine, which indicated an increase in stress, a tendency for HRV parameters (RMSSD, LF, HF and LF/HF) to decrease, and an increase in perceived stress after a 24h night-time on-call shift, suggesting a tilting of the sympathovagal balance towards increasing sympathetic activity. However, a study conducted at a German hospital with a sample of 20 medical residents found no significant difference after night-time shifts in the scores on attention tests or in measurements of HRV or biomarkers such as cortisol, epinephrine and norepinephrine [19]. In that study, significant differences were only found in the thyroid stimulating hormone (TSH), which indicates an increase in stress.

Nevertheless, as previously mentioned, not all studies have found an increase in sympathetic activity after on-call shifts among healthcare workers. Specifically, there are discrepancies regarding the evaluated parameters. For example, Harbeck et al. [19], in a sample of 20 doctors after 24h of being on call, found no significant differences in HRV or in cortisol values. The study by Lin et al. [21] showed a reduction in the LF/HF ratio, which is consistent with less sympathetic activity, during on-call shifts; and an increase in HF right after finishing the on-call shift, which would indicate the same tendency in the vagal domain within the ANS balance.

The results of our study show that participants in on-call shifts present a significant decrease in RT and a significant increase in errors and in anticipated responses as compared to the pre-test values and the contrast group. These results are consistent with those obtained in PVT in a group of miners who worked rotating shifts [40], and with the results of other studies in the field of healthcare [13,17,19], in which sleep deprivation negatively affected the results for the PVT.

In addition, the findings of this research provide evidence that intensive 24h on-call shifts with sleep deprivation affect the mood of healthcare professionals, boosting feelings of depression, anxiety and, in particular, hostility. These results confirm our hypothesis and are consistent with the findings of previous studies that have indicated greater levels of anxiety and perceived stress in medical residents after on-call shifts [19,20]. Our results indicate that besides levels of anxiety and stress, 24h on-call shifts with sleep deprivation would also affect other emotional variables, worsening mood and generating feelings of hostility, at least in a transitory manner. In addition to the impact that this could have on the mental health and quality of life of medical residents, it is important to consider that the psychological variables are associated in the post-test with impaired cognitive performance. Specifically, lower levels of happiness and higher levels of anxiety were associated with lower RT, and greater levels of hostility with more errors and lapses.

A secondary objective of this study consisted of examining the consistency among the different dimensions of the stress response. The results show moderate to strong correlations between several variables belonging to different domains. Furthermore, the pattern of correlations obtained from the pre-test was very similar to that obtained from the post-test, with certain exceptions. Specifically, the degree of association of SS with S/PS and hostility increased in the post-test. In addition, the HRV parameters and cortisol showed higher and significant correlations with the three indicators of cognitive performance. These results are in line with the model by Thayer and Hall [41] on dynamic behaviour systems. In accordance with this perspective, the endocrine system and the ANS are synchronised with one another, especially in response to high stress factors or substantial threats.

The previous literature contains far fewer studies that have explored the association between the variables based on correlations than those making comparisons using inferential statistics. Existing data from laboratory experiments with adults suggests a weak association between endocrine variables via activation of the hypothalamic pituitary-adrenal (HPA) axis and the response by the ANS with regard to the magnitude of the changes in response to stressful factors [42]. Our study, which was conducted in an ecologically valid context, in which the same participants are exposed to highly stressful daily situations, can result in a greater association between the response of the HPA axis and the ANS [43]. In fact, these same authors’ results from a study with 301 nurses doing night-time on-call shifts were similar to the results of our study, in which a strong correlation was observed between salivary cortisol and HRV. Other studies also examined the correlations between different variables during sleep deprivation, revealing mixed results. For example, Songy et al. [44] found a moderate correlation between the level of cortisol and components of mental health, such as mania in soldiers. In contrast, Guo et al. [45] found no correlation of any kind between biomarkers related to the hypothalamic-pituitary-thyroid (HPT) axis and burnout in nurses. The previous results suggest that the consistency between the hormonal system and ANS in response to stressful daily factors is closely related to individual responses and that a certain degree of synchrony only occurs in highly stressful situations in which cortisol increases above the normal circadian value.

## Conclusions

Our study shows deterioration in the physiological condition, mood and cognitive performance of doctors after a 24h on-call shift. The reduction in HRV supports the hypothesis that ANS disorders can influence the relationship between job stress and cardiovascular diseases, while at the same time it can lead to a greater tendency to make mistakes on the job. However, future studies should examine in detail the extent to which alterations of the ANS mediate in the relationship between exposure to prolonged work shifts and the risk of cardiovascular diseases. It is possible that doctors might eventually adapt to working in situations with a lack of sleep, but findings from this and other previous studies suggest the need to review medical on-call systems to reduce the stress load that has a direct effect on the working conditions.

This study has some limitations that must be taken into account. The control group has a significantly lower number of individuals than the experimental group due to problems in the recruitment of the sample and the availability of resident physicians. It is a problem inherent to research with a control group, but it is necessary to report it, as it could have an influence on the statistical results and the conclusions of the study.

## Supporting information

S1 File STROBE checklist of the study. (DOCX)

S2 File Clinical studies checklist. (DOCX)
